# *Calceolariaflavida* (Calceolariaceae) a new endemic species to central Chile

**DOI:** 10.3897/phytokeys.185.71755

**Published:** 2021-11-17

**Authors:** Nicolás Lavandero, Ludovica Santilli, Fernanda Pérez

**Affiliations:** 1 Departamento de Ecología, Facultad de Ciencias Biológicas, Pontificia Universidad Católica de Chile, Santiago, Chile Pontificia Universidad Católica de Chile Santiago Chile; 2 Museo Nacional de Historia Natural, Área Botánica, Interior Parque Quinta Normal S/N, Casilla 787, Santiago, Chile Museo Nacional de Historia Natural, Área Botánica Santiago Chile

**Keywords:** Chilean Mediterranean hotspot, Cerro el Roble, endemism, montane flora, South America, taxonomy

## Abstract

A new species of Calceolariasect.Cheiloncos endemic to central Chile is described. A comparison with the morphologically similar species *Calceolariaasperula* and *Calceolariapetioalaris* is made, and a key as well as detailed images to differentiate them is provided. The species is only known from the Natural Sanctuary Cerro El Roble, which is part of the coastal mountain range of central Chile and can be considered as Critically Endangered (CR) under the IUCN categories and criteria.

## Introduction

*Calceolaria* Linnaeus is the largest genus within Calceolariaceae with approx. 250 species distributed from Mexico to Southern Chile and Argentina (Molau 1988; Cosacov et al. 2009). The centre of diversity of the genus is found in Peru (Molau 1988). The genus includes herbs and shrubs characterised by opposite leaves and bilabiate corollas with a saccate lower lip with an infolded lobe normally bearing the elaiophore, a highly specialised, oil-producing structure involved in pollination (Vogel 1974).

The latest and most comprehensive revision of *Calceolaria* for Chile since Reiche (1911), published by Ehrhart (2000), was followed by the publication of new species segregated from the *C.integrifolia* complex (Ehrhart 2005), the revision of CalceolariasectionCalceolaria (Puppo and Novoa 2012) and by *Calceolariaphilippii*Eyzaguirre (2014). In Chile, there are 61 currently recognized species of *Calceolaria*, ten of which are further separated in a total of 30 subspecies (Rodriguez et al. 2018). Out of a total of 81 taxa, 60 (74%) are endemic to Chile. *Calceolaria* in Chile presents a wide distribution, from the latitudes of Arica y Parinacota region (18°35'S) to Magallanes region (54°50'S), and from the coast to the high elevations of the Andes (0–4300 m). Unsurprisingly, the area of most diversity is central Chile, from the Coquimbo region to the Araucania region (Ehrhart 2000). High levels of endemism in plants are common in the biogeographic area of central Chile which is recognized as a biodiversity hotspot (Myers et al. 2000; Arroyo et al. 2004).

Infrageneric classification within *Calceolaria* has been a subject of several works (Bentham 1846; Wettstein 1891; Kränzlin 1907; Pennell 1951). Molau (1988), working on the monograph of the tropical species of *Calceolaria*, restructured previous classifications and divided *Calceolaria* into three subgenera. CalceolariasubgenusCalceolaria comprises mainly species found in tropical regions, while CalceolariasubgenusCheiloncos (Wettstein) Pennell and CalceolariasubgenusRosula (Descole & Borsini) Molau comprise mostly temperate species. Subsequently, Ehrhart (2000), organized the species native to Chile in four sections: Calceolariasect.Calceolaria (one species, CalceolariapinnataL.subsp.pinnata), Calceolariasect.Kremastocheilos Witasek (one species, *Calceolariauniflora* Lam.), Calceolariasect.Tenella C. Ehrhart (one species, *Calceolariatenella* Poepp. & Endl.), and Calceolariasect.Cheiloncos Wettstein (47 species). The latter section, comprising most of the species present in Chile, was further divided into 14 informal Greges, based on vegetative and reproductive characters. More recently, molecular (Andersson 2006) and combined molecular and morphological studies (Cosacov et al. 2009), confirmed the subgeneric classification and some of the sections proposed by Molau (1988) as monophyletic, while most of the sections were found to be polyphyletic, and sections *Tenella* and *Kremastocheilos* sensu Ehrhart (2000) had little support. Due to the lack of resolution and the low sampling of Chilean species in Andersson (2006) and Cosacov et al. (2009), the classification proposed by Ehrhart (2000), particularly for Calceolariasect.Cheiloncos, is yet to be supported by studies with more extensive sampling.

The aim of this work is to describe a new species of *Calceolaria*, endemic to central Chile, assess its conservation status and provide a key for correct identification.

## Methods

**Figure 1.**
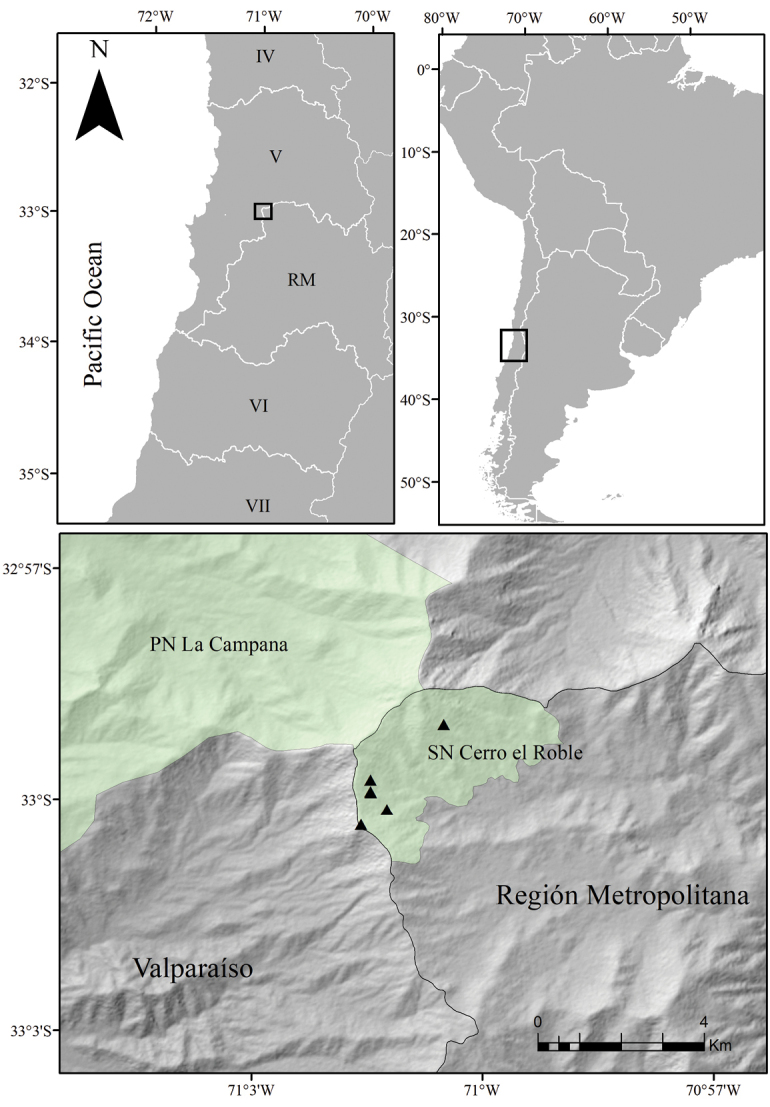
Distribution map of *Calceolariaflavida* (triangles) in Chile. Roman numbers represent administrative regions (IV Coquimbo, V Valparaiso, RM Metropolitan Region, VI O’Higgins, VII Maule). Green polygons represent protected areas (La Campana National Park and Natural Sanctuary Cerro El Roble).

Between the austral Spring of 2018–2020, several botanical explorations were carried out in the coastal mountain range of central Chile, between the limits of Valparaiso region and the Metropolitan region, in the Natural Sanctuary “Cerro El Roble”, 75 km northeast of Santiago’s urban area (Fig. 1). Specimens of *Calceolaria* that could not be assigned to any of the described species of the genus were found flowering in two sites close to the summit (1722–1729 m and 1766 m). The climate of the study site is classified as Mediterranean type with a rainfall regime characterized by an annual mean precipitation of 656 mm, a water deficit of 897 mm, and a 7-month dry season (Donoso et al. 2010). The soil is mainly composed of weathered granitic rocks (Brüggen 1950). The vegetation of this area is characterized by a relict deciduous forest dominated by *Nothofagusmacrocarpa* (Nothofagaceae), and surrounded by sclerophyllous forest and scrub of *Quillajasaponaria* (Quillajaceae) and *Lithraeacaustica* (Anacardiaceae) (Latorre-Beltrán 2012). At lower elevations, on the bottom of creeks with permanent flooding by groundwater, dense swamp forests of *Drimyswinteri* (Winteraceae) and *Lumachequen* (Myrtaceae) can be found. At the summit, a relict andean scrub dominated by *Chuquiragaoppositifolia* (Asteraceae) and *Azorellaprolifera* (Apiaceae) is found (Ministerio del Medio Ambiente 2018).

Specialised literature on systematics and taxonomy of *Calceolaria* was consulted (Witasek 1905; Valenzuela 1969; Ehrhart 2000; Ehrhart 2005). Herbarium specimens were collected and deposited at SGO (Lavandero 372; Lavandero & Santilli 201027). A systematic examination of selected specimens of *Calceolaria* found at SGO, EIF, CONC, as well as online digital images of specimens available on E, PH and US (acronyms following Thiers 2021) was carried out to search for more collections that could be morphologically coincident with the species. Herbarium specimens with similar morphology were found at SGO identified as *Calceolariaasperula* and Calceolariaaff.asperula. A thorough examination and dissection of the type specimen of *Calceolariaasperula* Phil. (SGO 055831) was performed, due to discrepancies with the schematic representation of the flower and the description of the species by Ehrhart (2000) and to confirm the identity of the new species.

The description and key were prepared after examining all available specimens. Description was made based on terminology following Ehrhart (2000) and Ehrhart (2005).

The assessment of the conservation status of the species was made using the International Union for Conservation of Nature (IUCN 2017) criteria. The extent of occurrence (EOO) and area of occupancy (AOO) were calculated using GeoCat (Bachman et al. 2011).

## Results

Following the morphological comparison of the plant collected with the specimens found in the consulted herbaria, we reached the conclusion that the individuals found in Cerro El Roble represent a new species. The new species is vegetatively similar to *Calceolariaasperula* Philippi and to *Calceolariapetioalaris* Cavanilles, both species endemic to Central Chile, belonging to Calceolariasect.Cheiloncos, group B, Grex X (C. dentatae) and Grex XI (C.petioalaris) sensu Ehrhart (2000), respectively. The three species have in common the growth form and other vegetative characters. They are perennial herbs with a lignified base, without any woody shoots aboveground that last from one growing season to the next one, with non-branching shoots aboveground, new shoots early in the season with very short internodes, giving them the appearance of a rosette-like structure (these internodes elongate later in the season and the rosette disappears), and ovate leaves and serrate margins, covered in glandular hairs.

**Figure 2.**
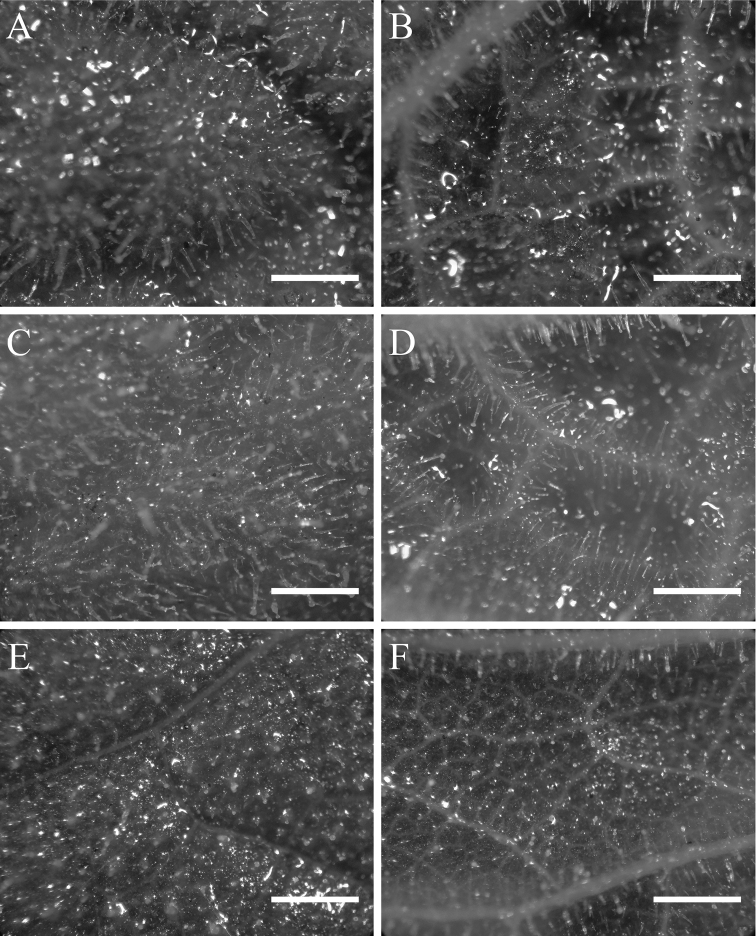
Indumentum type in leaves of *Calceolaria***A, C, E** upper leaf surface **B, D, F** lower leaf surface **A, B***Calceolariaasperula* (Lavandero 409 (SGO)) **C, D***Calceolariaflavida* (Lavandero & Santilli 201027 (SGO)) **E, F***Calceolariapetioalaris* (B. Rosende s/n). Scale bar: 1 mm.

**Figure 3.**
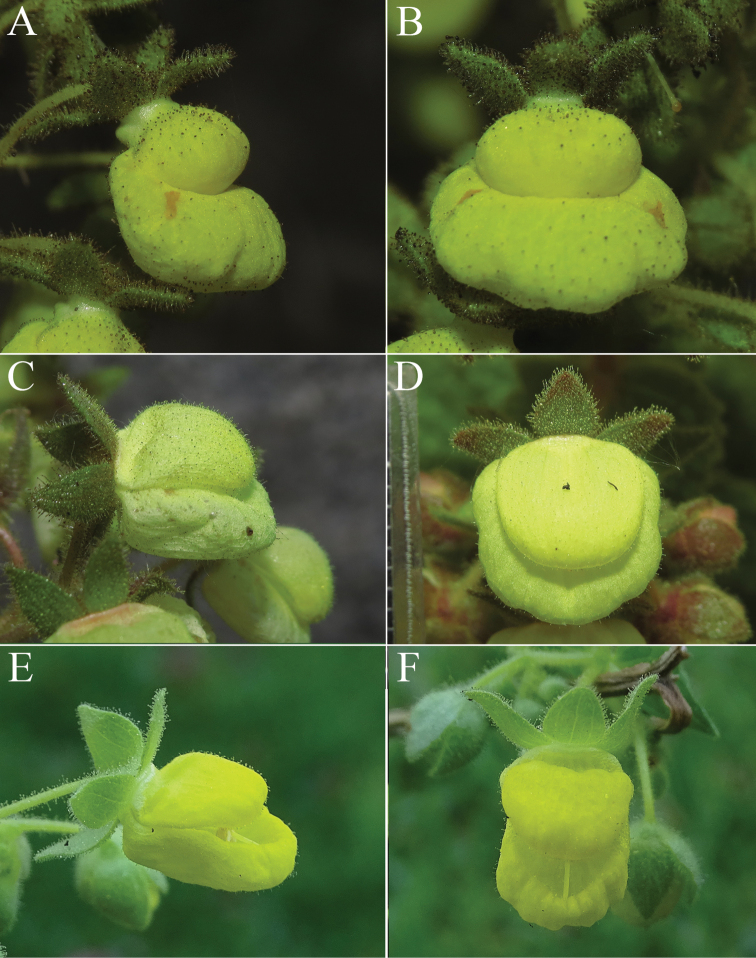
Lateral and frontal view of flowers (from left to right) of *Calceolaria***A, B***Calceolariaasperula* (Lavandero 409 (SGO)) **C, D***Calceolariaflavida* (Lavandero & Santilli 201027 (SGO)) **E, F***Calceolariapetioalaris* (B. Rosende s/n).

**Figure 4.**
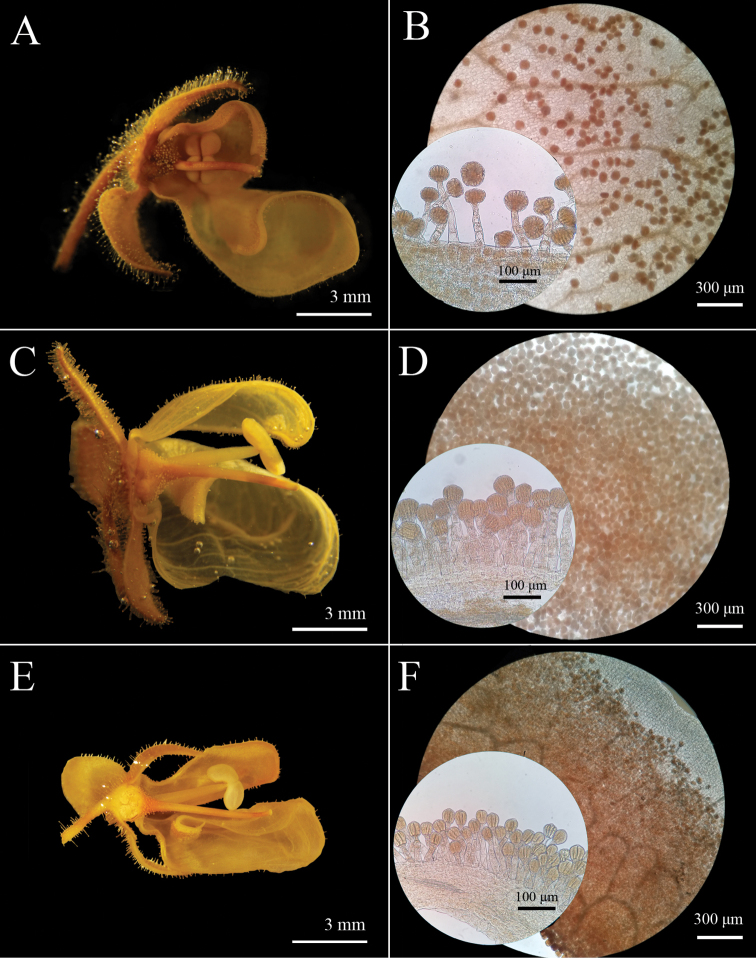
Lateral cross-section view of flowers of *Calceolaria* and detail of elaiophores **A, B***Calceolariaasperula* (Lavandero 409 (SGO)) **C, D***Calceolariaflavida* (Lavandero & Santilli 201027 (SGO)) **E, F***Calceolariapetioalaris* (B. Rosende s/n).

Nevertheless, both leaf texture and indumentum and flower morphology differ considerably among the three species (Figs 2–4). The secondary and tertiary venation of the new species is visibly impressed on the adaxial side and prominent on the abaxial side of the lamina (Fig. 2C–D). The leaf indumentum is formed by long and densely arranged glandular and eglandular trichomes, which gives a glutinous and sticky texture. Freshly collected material can hardly be separated from the paper in which it is dried. The leaf texture and indumentum is similar to *Calceolariaasperula* (Fig. 2A–B), but the latter has a deeply impressed venation on the upper surface, forming deep cavities, giving the most rugose aspect of the three species. *Calceolariapetioalaris*, has a venation slightly impressed on the adaxial side and slightly prominent on the abaxial side, with leaf indumentum composed of short glandular and eglandular trichomes, which give a less glutinous and sticky texture; freshly collected material can easily be separated from the paper in which it is dried (Fig. 2E–F). The flower lips of the new species are rounded in shape, saccate, and the upper lip is narrower and longer than half the length of the lower lip (Fig. 3C–D), while the flower lips of *C.petioalaris* are squared, flat and almost equal in width, and the upper lip is shorter than the lower lip (Fig. 3E–F). The length of the stamens of the new plant and *C.petioalaris* is similar, while *C.asperula* presents much shorter filaments (Fig. 4A, C, E). The new species shows an elaiophore similar to *C.petioalaris* and different from the one of *C.asperula* which has an elaiophore made of dispersed oil producing trichomes (Fig. 4B, D, F), a character that is unique among *Calceolaria* found in Chile (Ehrhart 2000).

The dissection of the type specimen of *Calceolariaasperula* (SGO 055831) showed that the lips differ in size, being the upper lip less than half the size of the lower lip (Suppl. material 1). This contrasts with the schematic representation of the flower of *Calceolariaasperula* found in Ehrhart (2000). The dissection also confirms that the elaiophore of *Calceolariaasperula*, is formed by dispersed oil producing trichomes (Suppl. material 1: Fig. S1D–E).

### Taxonomic treatment

#### Calceolaria flavida

Taxon classificationPlantaeLamialesCalceolariaceae

Lavandero & Santilli sp. nov.

77B1E4E5-BE99-5DAB-964F-94597F3AB7ED

urn:lsid:ipni.org:names:77222669-1

**Figure 5.**
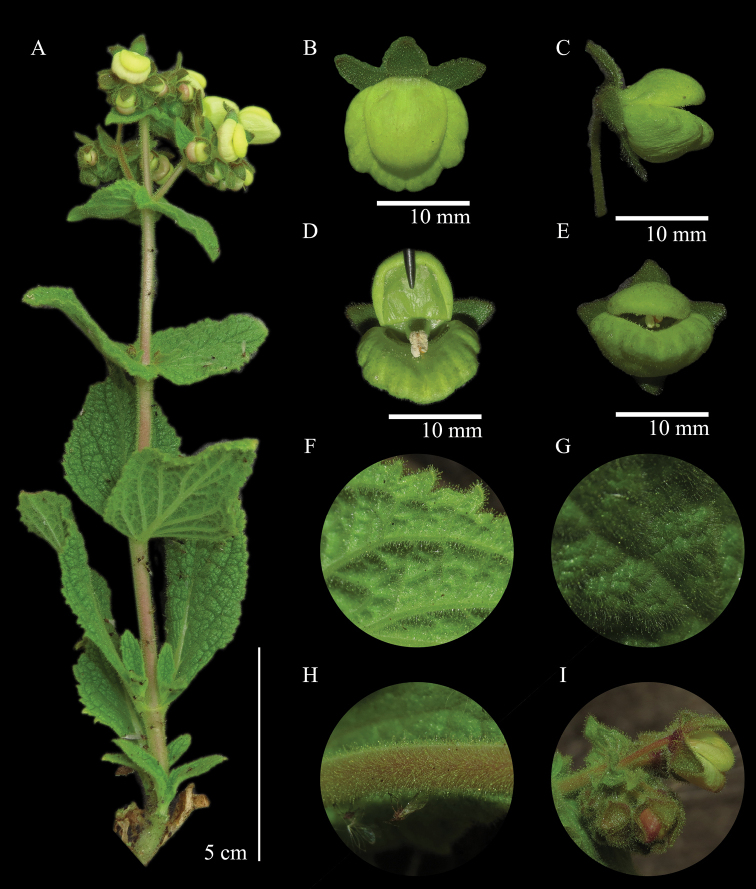
*Calceolariaflavida***A** habit **B** upper side view of flower **C** lateral view of flower **D** frontal view of flower with upper lip open **E** frontal view of flower **F** detail of abaxial side of leaf **G** detail of adaxial side of leaf **H** detail of stems **I** detail of early-flowering inflorescence.

**Figure 6.**
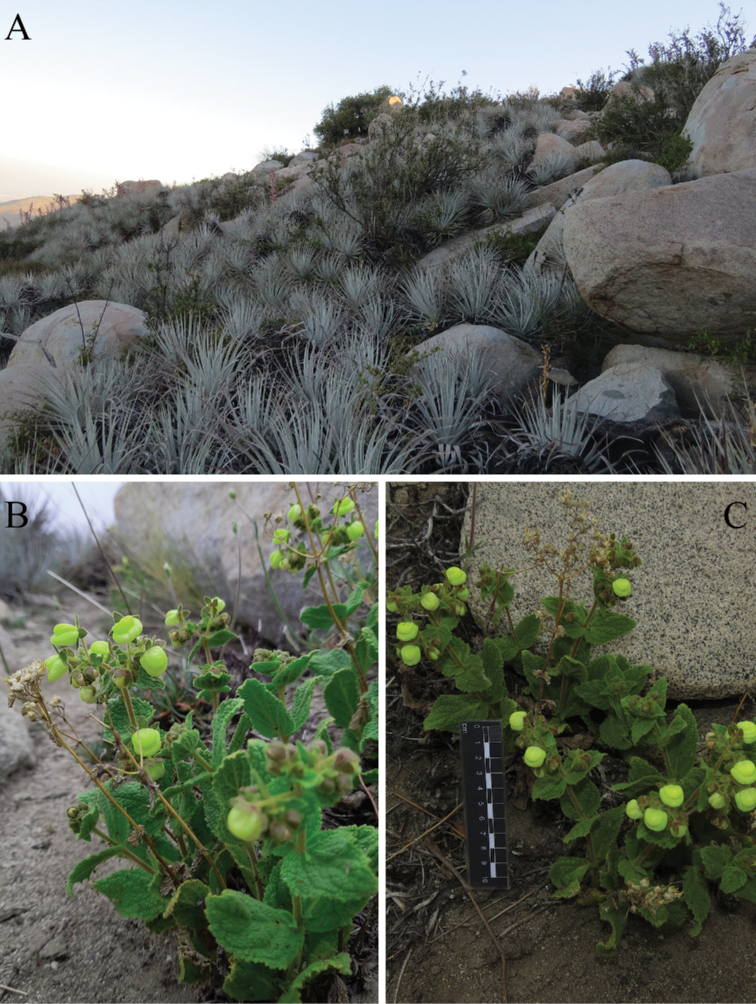
Habitat of *Calceolariaflavida***A** NW-facing slopes dominated by *Puyacoerulea var. coerulea*, *Lithraeacaustica* and *Gochnatiafoliolosa* (Natural Sanctuary Cerro El Roble, Región Metropolitana, Chile) **B, C** habit of *Calceolariaflavida*.

Figures 2C–D , 3C–D , 4C–D , 5 , 6B-C

##### Diagnosis.

*C.flavida* is most similar to *C.asperula* and *C.petioalaris* in growth habit and in having leaves of similar shape covered in glandular hairs. *C.flavida* can easily be distinguished from *C.asperula* in having pale yellow corolla (vs. bright yellow), the upper lip longer than half the length of the lower lip (vs. upper lip shorter than half the length of the lower lip), anthers much shorter than filaments and opening towards the distal part of the upper lip (vs. anthers as long as filaments and opening toward the style) and an elaiophore with densely arranged oil-producing trichomes (vs. dispersed oil producing trichomes). It can be distinguished from *C.petioalaris* by its reddish stems (vs. green), secondary and tertiary veins of the adaxial side of leaf lamina visibly impressed (vs. secondary and tertiary veins of the adaxial side of leaf lamina slightly impressed), pale yellow corolla (vs. bright yellow), upper lip narrower than lower lip seen from above (vs. upper lip as wide as lower lip), lips rounded in shape (vs. squared), saccate upper lip (vs. flat), and style inserted in corolla (vs. exserted).

##### Type.

Chile. Región Metropolitana, Cerro El Roble, 1674 m, 32°59'54" S - 71°01'27" W, 17-12-2006, *N. García & M. Muñoz 3836* (holotype SGO 157641!)

##### Description.

***Perennial*** herb up to 60 cm; base lignified, growth form type 6 sensu Ehrhart (2000). ***Stems*** reddish, erect, lower vegetative part not branched, densely covered with glandular hairs accompanied by much longer regular hairs; internodes very short at the beginning of the growing season, giving the aspect a rosette, these internodes extend throughout the growing season, being progressively longer towards the apex; stems renewing from the lignified base every season. ***Leaves*** opposite, green; lower leaves lanceolate, petiolate, base cuneate, apex acute; upper leaves ovate, sessile to partially amplexicaul, base subcordate, apex acute; (1.7–)2–7(–8.5) × (1.2–)2.5–3.5(–4) cm, margins serrate or slightly biserrate, lamina hirsute, trichomes glandular; venation impressed in the upper surface and prominent in the lower surface, secondary and tertiary veins of the adaxial side of leaf lamina visibly impressed. ***Synflorescence*** not conspicuously elevated from the vegetative part, up to 32 cm tall including the basal internode of the main florescence; basal internode 46–85 mm and as long as the internodes between the leaves at most; main inflorescence composed of 1–3 pairs of 15–19-flowered cymes; hypopodia 3.4–6.4 cm; pedicels 6.5–10.2(–20) mm; cyme bracts sessile, 14–30 × 8–25 mm, subordinate bracts sessile, 5–9 × 3–6 mm. ***Sepals*** green, ovate, 6.5–7.3 × 3.7–4.2 mm, densely covered in glandular hairs on both sides. ***Corolla*** pale yellow, evenly covered in glandular hairs, longitudinal axes of the lips parallel to each other, the upper lip longer than half the length of the lower lip and close to one another; lower lip saccate, rounded and lobed, 9.0–10.5 (length) × 9.2 (width) × 6.0–6.2 (height) mm; aperture narrow and oval, facing the upper lip, depression of the upper side almost absent; upper lip saccate, rounded to truncate seen from above, narrower than lower lip seen from above, 6.8–9.3 × 8.0–9.2 × 4.0–4.1 mm; aperture wide and almost reaching the sides of the lip. ***Elaiophore*** type 1 (sensu Ehrhart 2000), same length as the opening of the lower lip, 7.6 × 2.4 mm, folded inwards into the lower lip and covering the end of the lobe; oil-producing trichomes 190–245(-270) μm long, stalk generally (3–)4–6(–7)-celled and glandular head 38–44-celled, densely arranged, forming a well-defined and compact cushion. ***Stamens*** 2, included in the upper lip, stamens and style almost parallel, forming an acute angle; filaments 5.1–5.4 mm; anthers shorter than filaments, dithecal, basifixed, with line of dehiscence opening towards the distal part of the upper lip, 2.7–3.3 × 1.2–1.4 mm; ***Gynoecium*** (ovary + style) 6.0 mm; ovary densely covered by glandular hairs; style inserted in upper lip, 4.3 mm; stigma inconspicuous. ***Capsule*** conic, acuminate, 5.3–5.6 × 3.4–3.7 mm, with sparse glandular hairs. ***Seeds*** globose, 520–600 × 280–340 μm, seed surface type 3 (sensu Ehrhart 2000).

##### Habitat and distribution.

*C.flavida* seems to be endemic to the Natural Sanctuary Cerro El Roble (33°00'S 71°01' W), which is part of the coastal mountain range of central Chile (Fig. 1). It can be found on slopes with N-NW orientation at elevations of 1450–2200 m. *Calceolariaflavida* grows on soils of granitic origin, between rocks in open areas within sclerophyllous scrub dominated by *Puyacoerulea* Lindl. *var. coerulea* (Bromeliaceae), *Lithraeacaustica* (Molina) Hook. & Arn. and *Gochnatiafoliolosa* (D. Don) D. Don ex Hook. & Arn (Asteraceae) (Fig. 6A).

##### Phenology.

The species was found flowering between October and January.

##### Etymology.

The specific epithet *flavida* is a singular, feminine, nominative Latin adjective alluding to pale yellow colour of corolla.

##### Conservation status.

*C.flavida* can be considered as Critically Endangered (CR) under the IUCN categories and criteria B1ab(iii). The criterion B1 was selected because its extent of occurrence is < 100 km^2^ (0.995 km^2^). The criterion “a” was selected because it is known to exist at only one location (=1). The criterion “b(iii)” was selected because there is a projected decline in the area, extent and quality of habitat. Climate change and the persistent drought that has been affecting Central Chile represent a threat to plants that grow in the region. Starting in 2010, the Chilean territory between the Coquimbo and Araucanía Regions has experienced a rise in temperature and a precipitation deficit of approximately 30% causing visible deterioration of non-irrigated vegetation as well as increasing the likeability of forest fires (Garreaud, 2015). The species grows within the Natural Sanctuary Cerro El Roble.

##### Additional specimens examined.

**Chile. Región Metropolitana**: Provincia de Chacabuco: Caleu, Cerro El Roble, antes de los potreros, 12 January 2002, *N. García 3863* (EIF); Cerro El Roble, km 5 camino a la cumbre, 1 January 2003, *A. Moreira 863* (SGO); Subida a Cerro El Roble, poco más abajo Portezuelo Rauco, 27 October 2005, *M. Muñoz 4741* (SGO); Caleu, camino a El Roble, 1 km más abajo del corral, 17 December 2006, *N. García & M. Muñoz 3839* (SGO); Cerro El Roble, arriba del refugio a 3.5 km desde la entrada, 29 November 2019, *N. Lavandero 372* (SGO); Cerro El Roble, 27 October 2020, *Lavandero & Santilli 201027* (SGO).

### Key of herbaceous *Calceolaria*

Key of herbaceous *Calceolaria* with a woody base and glandular indumentum, taller than 20 cm, with leaves along the stem, not arranged in a rosette, entire with tendency to be sessile, less than five times longer than wide, longer than 25 mm (replace couplet 17 of Ehrhart’s key to *Calceolaria* of Chile (Ehrhart 2000)).

**Table:** 

1	Leaves with secondary and tertiary veins deeply impressed on the adaxial side; glandular hairs dark, scattered along the corolla; upper lip less than half the length of the lower lip, anthers as long as filaments, and opening toward the style, elaiophore with scattered oil-producing hairs	***C.asperula***
–	Leaves with secondary and tertiary visibly or slightly impressed on the adaxial side; glandular hairs clear, densely arranged along the corolla; upper lip more than half the length of the lower lip, anthers much shorter than filaments and opening towards the distal part of the upper lip, elaiophore with densely arranged oil-producing hairs	**2**
2	Stems green; leaf margin dentate with sharp teeth, indumentum of short glandular hairs; leaves with secondary and tertiary veins slightly impressed on the adaxial side; corolla bright yellow, upper lip as wide as lower lip seen from above, lips squared in shape, bright yellow, flat; style exserted from corolla	***C.petioalaris***
–	Stems reddish; leaf margin dentate, with smooth teeth, indumentum of long glandular hairs; leaves with secondary and tertiary veins visibly impressed on the adaxial side; corolla pale yellow, upper lip narrower than lower lip seen from above, lips rounded in shape, pale yellow, saccate; style inserted in corolla	***C.flavida***

## Discussion

Initial confusion existed regarding the identity of *Calceolariaasperula*. In the protologue of *C.asperula*, Philippi (1895) only gives the diameter of the inferior lip in a short description without mentioning the upper lip. Ehrhart (2000) only illustrates the taxon with a schematic representation of the flower, showing lips of almost equal size, being the upper lip slightly smaller than the lower, and only gives the size of the upper lip seen from above, being 5.5 mm in diameter approximately. Ehrhart (2000) describes it as a species with a unique combination of characters such as the anthers opening towards the style and an elaiophore made of dispersed oil-producing hairs. The dissection of a flower from the type material of *C.asperula* (SGO055831) (Suppl. material 1) shows Ehrhart’s (2000) description to be mostly accurate regarding vegetative morphology and elaiophore structure, but the upper and lower lip description is incomplete and imprecise, making the schematic representation of the flower doubtful. This imprecision in Ehrhart’s schematic representation of the flower might explain why specimens of *Calceolariaflavida* found in SGO were formerly identified as *C.asperula* or, in some cases, as Calceolariaaff.asperula.

The classification proposed by Ehrhart (2000) for the species of *Calceolaria* present in Chile, although not yet confirmed by molecular evidence, is however very useful for grouping species based on both vegetative and reproductive characters, the latter having higher weight for the classification at lower levels. Within Calceolariasect.Cheiloncos, *Calceolariaflavida* falls into group B, by having an upper lip at least 1/3 as long as the lower lip and anthers shorter than the filaments. At the Grex level, it could be classified within two Greges, Grex X (comprising *C.densifolia* Phil., *C.dentata* Ruiz & Pav., *C.flavovirens* C.Ehrhart, *C.lepida* Phil., *C.morisii* Walp., *C.nitida* Colla, *C.polifolia* Hook, *C.asperula* Phil. and *C.purpurea* Graham) and Grex XI (comprising *C.petioalaris* Cav. and *C.latifolia* Benth.). Among these species, *Calceolariaflavida* has clear affinities with two species, *C.asperula* and *C.petioalaris*, based on leaf shape and growth habit, leaving out all the other species within these two Greges presenting a shrubby habit. The most useful characters to differentiate the new species from the morphologically most similar species *C.asperula* and *C.petioalaris*, proved to be flower related, showing the importance of these stable characters for the taxonomy of *Calceolaria*. From the ecological and geographical perspective, these three species can be clearly distinguished. *Calceolariapetioalaris* is the only one among the three species that associates with meso-hydrophytic conditions, growing most of the time near water courses such as small streams or ravines from the coast up to mid-elevations of the Andean cordillera (50–1800 m), between the Coquimbo and Maule Regions (Ehrhart 2000). *Calceolariaasperula* can be found in more xeric conditions at elevations between 800–2000 m, in both Coastal and Andean Cordilleras, in open and rocky areas among the sclerophyllous montane vegetation, between the Metropolitan and O’Higgins regions. *Calceolariaflavida* is more similar to *C.asperula* in terms of its ecology. It can also be found in xeric conditions associated with sclerophyllous vegetation at mid-elevations (1450–2200 m), but only grows on soils of granitic origin with N-NW orientation of Cerro El Roble, one of the tallest peaks of the Coastal Cordillera of Central Chile.

Out of 81 taxa recognized for Chile, 61 are endemic (Rodriguez et al. 2018; RBG Edinburgh 2021). Most of the endemism is located around Coquimbo and Maule Regions (29°02'S–36°32'S), being Valparaiso and the Metropolitan Region (32°01'S–34°17'S) the most species-rich regions (Rodríguez et al. 2018; RBG Edinburgh 2021). The Coastal cordillera of central Chile is already known to host several species of *Calceolaria* endemic to Chile (Ehrhart 2000; Ehrhart 2005; García 2010; Flores-Toro and Amigo 2013). Moreover, Cerro El Roble hosts two narrow-endemic (Sensu Molau 1988) species of the genus: *Calceolariacaleuana* Muñoz-Schick & Moreira, found on the summit of Cerro El Roble and another locality in the limits of Valparaiso and Coquimbo Regions (Muñoz-Schick and Moreira-Muñoz 2008; Muñoz-Schick and Moreira-Muñoz 2009), and CalceolariaascendensLindl.subsp.exigua (Witasek) Nic. García, a rupicolous taxon found only on rocky outcrops of the coastal Cordillera between 32°42'S–33°12'S at elevations of 1600–2100 m (García 2010). La Campana National Park, adjacent to Natural Sanctuary Cerro El Roble, is also home to the narrow endemic *Calceolariacampanae* Phillipi, which grows between the rock crevices near the summit of Cerro La Campana. This pattern of diversity found in *Calceolaria* is not unusual for the Chilean flora. Several genera share the same pattern of high diversity and endemism in Central Chile, such as *Senecio* L., *Chaetanthera* Ruiz & Pav., *Haplopappus* Cassini, *Leucheria* Lagascae, *Oxalis* L. and *Adesmia* D.C. (Arroyo et al. 1995; Fuentes et al. 1995). A combination of high climatic heterogeneity due to latitudinal and altitudinal gradients (Armesto et al. 2007), plus the climatic history of the Quaternary, particularly glaciations and the presence of coastal refugia, are the probable drivers for the higher diversity and endemism in this region (Arroyo et al. 1995; Villagrán 1995; Hinojosa and Villagrán 1997; Villagrán and Hinojosa 1997).

The origin and present distribution of *C.flavida* could be related to the series of expansions/contractions and isolation of the vegetation belts in the Coastal Cordillera due to the glacial/interglacial cycles. Since there is no updated phylogeny of *Calceolaria*, no relationships could be inferred for *C.flavida*. Based on its morphology and following the preliminary phylogenetic studies (Cosacov et al. 2009), it could be hypothesized that it belongs to the subgenus Cheiloncossect.Rugosae along with the most morphologically similar species *Calceolariaasperula* and *Calceolariapetioalaris*, all endemic to central Chile. A well-resolved phylogeny of the genus could help clarify the relationships among these species and establish a better understanding of the complex evolutionary history of *Calceolaria* in central Chile.

Since *Calceolariaflavida* appears to be a narrow-endemic and our preliminary assessment classifies it as Critically Endangered (CR), further surveys in the Coastal Cordillera of central Chile are needed in order to fully understand its distribution and population size.

### Additional specimens examined

*Calceolariaasperula*. **Chile. Región Metropolitana**: Provincia de Melipilla: Reserva Nacional Roblería del Cobre de Loncha, 18 November 2004, *P. Baxter*, *F. Bustos*, *M.F. Gardner*, *P. Hechenleitner V. & P.I. Thomas 1439* (SGO!, E[photo]!); Reserva Natural Altos de Cantillana, sendero desde refugio el alto a Horcón de Piedra, 28 December 2019, *N. Lavandero 671* (SGO!); Reserva Natural Altos de Cantillana, sendero desde refugio el alto a Horcón de Piedra, 20 November 2020, *N. Lavandero 201120* (SGO!); Provincia Cordillera: Pirque, Reserva Nacional Río Clarillo, Sendero Quebrada Jorquera, 29 January 2013, *T. Christian*, *M.F. Gardner & V. Morales 343* (E [photo]!); Reserva Nacional Río Clarillo, Cajón de los Cipreses, 13 December 2019, *N. Lavandero 409* (SGO!); **Región de O’Higgins**: Cajón de los Cipreses, March 1875, *R.A. Philippi s.n.* (SGO 055831!, PH[photo]!), Provincia del Cachapoal: Rancagua, road from Coya to Mina La Juanita, ca. 8.3 km above retén de Carabineros, 18 January 1995, *L.R. Landrum & J. Martínez 8486* (SGO!); Machalí, Road from Coya to Machalí, 3 January 2009, *M.F. Gardner & S.G. Knees 8453* (E [photo]!); Copada, open rocky slope, 25 January 1925, *F.W. Pennell 12272* (US [photo]!)

*Calceolariapetioalaris*. **Chile. Región de Coquimbo**: Provincia de Limarí: Camino a Mina Lapislazuli, 14 January 2009, *Fundación Philippi 349* (SGO); Provincia Choapa: Camino Tilama - cuesta Las Palmas, 18 November 2002, *A. Moreira 784* (SGO): Provincia de Elqui: Illapel, Cuzcuz, 5 November 1985, *M. A. Trivelli s.n* (SGO). **Región de Valparaíso**: Provincia de Petorca: Catemu al N entre Las Majadas y Campamento Cerro Negro, 28 December 2001, *A. Moreira 643* (SGO); Provincia Los Andes: Camino a Portillo, 11 December 2001, *M. Chamy & M. Piovano 15* (SGO); camino a Portillo, 11 December 2001, *M. Chamy & M. Piovano 16* (SGO); Ruta 60, Camino hacia Portillo 21 December 2019, *N. Lavandero 421* (SGO); Provincia de Quillota: Olmué, Parque Nacional La Campana, sector Granizo, 1 February 1998, *Baxter* et al. *s.n* (SGO); Parque Nacional La Campana, sector Granizo, Mina Pronosticada, 6 January 2001, *A. Moreira 510* (SGO); Parque Nacional La Campana, sector Granizo, Mina Pronosticada, March 2001, *A. Moreira 533* (SGO). **Región Metropolitana**: Provincia de Santiago: Hacienda Rinconada de Cerda, Maipú, Quebrada de la Plata, 1 October 1936, *C. Muñoz s.n* (SGO); Cajón del Estero La Leonera, ladera al NO, 27 February 2000, *Arroyo* et al. *201380* (SGO); Provincia Cordillera: Lo Valdés, 28 December 2000, *A. Moreira 498* (SGO); camino al Embalse del Yeso, 21 January 1995, *M. Muñoz & A. Moreira 3704* (SGO); Provincia de Melipilla: Alhué, Reserva Nacional Roblería del Cobre de Loncha, 18 November 2004, *Baxter* et al. *s.n* (SGO); Cuesta Zapata, 7 October 1988, *von Bohlen 512* (SGO), Cuesta Zapata, 4 July 1989, *von Bohlen 581* (SGO); Cuesta Zapata, 18 December 2001, *M. Chamy & M. Piovano 2* (SGO), Cuesta Zapata, 18 December 2001, *M. Chamy & M. Piovano 8* (SGO); Provincia de Chacabuco: Cuesta La Dormida, entre Cruce Caleu y Portezuelo, 4 May 2003, *A. Moreira 958* (SGO). **Región Libertador Bernardo O’Higgins**: Provincia de Colchagua, San Fernando, Sector La Rufina, Zona de Intersección Río Tinguiririca con Río Clarillo, 16 November 2004, *Baxter* et al *s.n* (SGO). **Región del Maule**: Provincia de Curicó: quebrada de los Pejerreyes en Los Queñes, 10 January 1968, *P. Aravena 354* (SGO)

## Supplementary Material

XML Treatment for Calceolaria flavida

## Supplementary materials

Supplementary material 1 Figure S1. Type specimen of *Calceolariaasperula* Phil. (SGO 055831) Data type Figure. Explanation note **A** complete specimen **B** detail of dried flower **C** detail of rehydrated flower **D** detail of the elaiophore **E** detail of the scattered oil-producing glands of the elaiophore. https://binary.pensoft.net/file/611523This dataset is made available under the Open Database License (http://opendatacommons.org/licenses/odbl/1.0/). The Open Database License (ODbL) is a license agreement intended to allow users to freely share, modify, and use this Dataset while maintaining this same freedom for others, provided that the original source and author(s) are credited.Nicolás Lavandero, Ludovica Santilli
