# Supplementary figures and images for: Calceolariaflavida (Calceolariaceae) a new endemic species to central Chile
Source: PhytoKeys. 2021 Nov 17;185:99–116. doi: 10.3897/phytokeys.185.71755 (PMC8613134; doi:10.3897/phytokeys.185.71755)

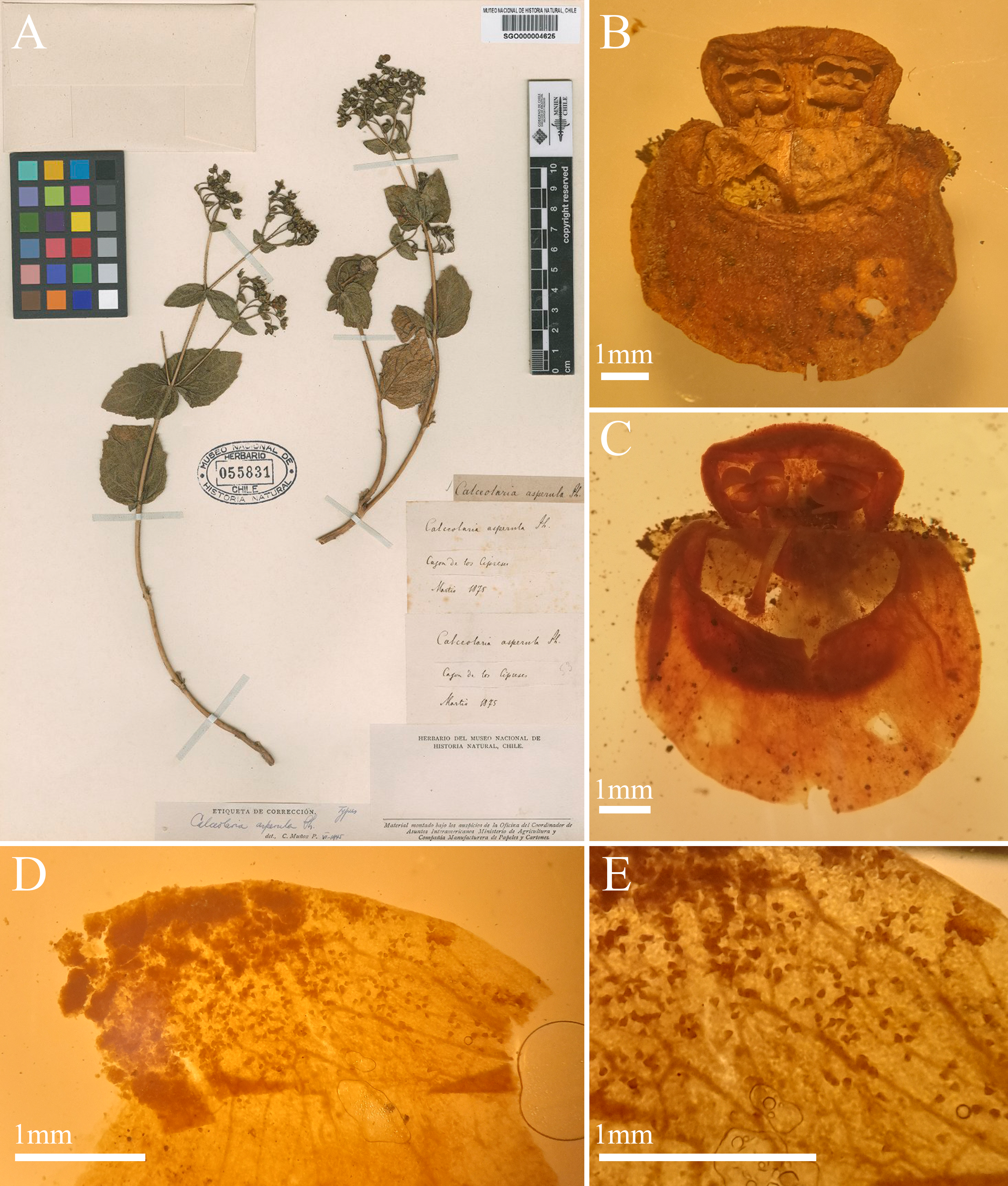

Supplement: Supplementary material 1 — Figure S1. Type specimen of Calceolariaasperula Phil. (SGO 055831) [file phytokeys-185-099-s001.tif]
